# Multivariate analysis of pedicle screw invasion of the proximal facet joint after lumbar surgery

**DOI:** 10.1186/s12891-021-04975-2

**Published:** 2022-01-06

**Authors:** Peng Tao Wang, Jia Nan Zhang, Tuan Jiang Liu, Jun Song Yang, Ding Jun Hao

**Affiliations:** Spine Surgery, Honghui Hospital Affiliated with Xi’an Jiaotong University, Xi’an City, 710054 Shaanxi Province China

**Keywords:** Lumbar surgery, Pedicle screw, Proximal facet joint, Multivariate analysis

## Abstract

**Background:**

To analyze the risk factors for pedicle screw invasion of the proximal facet joint after lumbar surgery.

**Methods:**

From January 2019 to January 2021, 1794 patients with lumbar degenerative disease, such as lumbar disc herniation, lumbar spinal stenosis and lumbar spondylolisthesis, were treated at our hospital. In all, 1221 cases were included. General data (sex, age, BMI), bone mineral density, proximal facet joint angle, degenerative lumbar spondylolisthesis, isthmic lumbar spondylolisthesis and fixed segment in the two groups were recorded. After the operation, vertebral CT of the corresponding surgical segments was performed for three-dimensional reconstruction and evaluation of whether the vertebral arch root screw interfered with the proximal facet joint. The included cases were divided into an invasion group and a noninvasion group. Univariate analysis was used to screen the risk factors for pedicle screw invasion of the proximal facet joint after lumbar surgery, and the selected risk factors were included in the logistic model for multivariate analysis.

**Results:**

The single-factor analysis showed a significant difference in age, BMI, proximal facet joint angle, degenerative lumbar spondylolisthesis, and fixed segment (*P* < 0.1). Multifactor analysis of the logistic model showed a significant difference for age ≥ 50 years (*P* < 0.001, OR = 2.291), BMI > 28 kg/m2 (*P* < 0.001, OR = 2.548), degenerative lumbar spondylolisthesis (*P* < 0.001, OR = 2.187), gorge cleft lumbar relaxation (*P* < 0.001, OR = 2.410), proximal facet joint angle (35 ~ 45°: *P* < 0.001, OR = 3.151; > 45°: *P* < 0.001, OR = 3.578), and fixed segment (lower lumbar spine: *P* < 0.001, OR = 2.912).

**Conclusion:**

Age (≥ 50 years old), BMI (> 28 kg/m2), proximal facet joint angle (35 ~ 45°, > 45°), degenerative lumbar spondylolisthesis, isthmic lumbar spondylolisthesis and fixed segment (lower lumbar spine) are independent risk factors for pedicle screw invasion of the proximal facet joint after lumbar surgery. Compared with degenerative lumbar spondylolisthesis, facet joint intrusion is more likely in isthmic lumbar spondylolisthesis.

**Supplementary Information:**

The online version contains supplementary material available at 10.1186/s12891-021-04975-2.

## Background

At present, in clinical practice, the pedicle screw system is often used for the internal fixation of corresponding vertebral bodies in the treatment of various diseases, such as spinal instability, vertebral fracture, lumbar degenerative disease and spinal deformity [1]. However, the implantation of pedicle screws may cause different degrees of damage to the proximal facet joint [2–6], which will increase the pressure on adjacent intervertebral discs and facet joints and affect the stability of the spine, thus increasing the relative displacement and angular deformity between adjacent vertebral bodies and increasing the possibility of degenerative diseases in adjacent segments. According to relevant reports in the literature [5, 7], the implantation of pedicle screws after lumbar surgery is an important risk factor for proximal facet joint invasion, but there have been few reports on the risk factors related to pedicle screw implantation. Therefore, this study used multivariate analysis to explore the risk factors for proximal facet joint invasion related to pedicle screw implantation.

## Materials and methods

### Inclusion and exclusion criteria

This study was a retrospective study approved by the medical ethics committee of our hospital. The inclusion criteria were as follows: ① lumbar degenerative disease, such as lumbar disc herniation, lumbar spinal stenosis, and lumbar spondylolisthesis; ② failure of conservative treatment for more than 3 months; ③ posterior lumbar surgery with internal fixation; and ④ manual screw placement. The exclusion criteria were as follows: ① spinal tumor, metastasis or hemangioma; ② previous history of lumbar surgery; and ③ congenital or traumatic spinal deformity.

### General information

According to the above criteria, 1221 patients with lumbar degenerative disease, such as lumbar disc herniation, lumbar spinal stenosis and lumbar spondylolisthesis, treated at our hospital from January 2019 to January 2021 were included, and 573 patients were excluded. According to Herren et al. [8], invasion was classified as follows: grade 0, the screw did not touch the joint; grade 1, the screw touched the joint; grade 2, the screw obviously entered the articular cavity; and grade 3, the screw damaged the medial wall of the articular cavity and the pedicle at the same time. The patients who met the criteria were divided into two groups: the noninvasion group, comprising patients with grade 0 invasion, and the invasion group, comprising patients with grade 1, 2 or 3 invasion. There were 724 patients in the invasion group, including 360/364 males/females, aged 24-87 years (63.18 ± 7.01). There were 497 patients in the noninvasion group, including 252/245 males/females, aged 34-83 years (64.27 ± 8.22). There were 523 cases of lumbar disc herniation, 324 cases of lumbar spinal stenosis, and 374 cases of lumbar spondylolisthesis. Screws were placed in the upper lumbar spine in 163 cases and the lower lumbar spine in 1058 cases.

### Therapeutic technique

After general anesthesia, the patient was placed in the prone position, and disinfection with amiloride was performed according to the routine procedure for lumbar posterior surgery, followed by draping with a sterile towel and the placement of a knife-edge thin film. Under C-arm guidance, a straight incision was made in the skin, subcutaneous tissue and fascia of the lower back layer by layer, followed by blunt dissection under the periosteum near the spinous process to expose the spinous process, lamina and bilateral articular processes of the vertebral body. According to the anatomical structure and the external position of the transverse process and lamina, the pedicle screw channel was created in the pedicle of the vertebral body to be fixed by hand, followed by placement of the positioning needle. After confirmation of satisfactory positioning of the needle under C-arm fluoroscopy, the screw was placed. If the position was poor, it was corrected. After the dural sac and nerve root were grasped, the fibrous ring was cut with a small sharp knife, and the intervertebral disc was treated with a reamer, curette and nucleus pulposus forceps, resulting in bleeding from the upper and lower endplates. After sufficient decompression, the nerve root canal was expanded, and relaxation of the nerve root was achieved after sufficient decompression. A suitable prebent longitudinal connecting rod was installed; the intervertebral space of the vertebral body affected by spondylolisthesis was properly opened, followed by lifting and opening for reduction. After the wound was washed and the model was tested, the bone particles obtained from intraoperative decompression were trimmed to an appropriate size and implanted in front of the intervertebral space. An intervertebral fusion cage filled with treated autologous bone particles was implanted in the rear, approximately 5 mm away from the posterior edge of the vertebral body. The screw was held properly during compression of the intervertebral space and then clamped tightly. The wound was rinsed with normal saline, and hemostasis was carefully achieved. After confirming that there was no active bleeding in the wound, a drainage tube was placed in the wound, and the wound was sutured layer by layer, pressed and bound with sterile dressing.

According to the grading method of Herren et al. [8], two senior surgeons evaluated whether the proximal facet joints of the lumbar spine were invaded through postoperative CT examination. If the views of the two doctors conflicted, a third senior chief physician was consulted for evaluation and determination of a clear conclusion.

### Observation indexes

According to the relevant literature reports and observation results, the following eight risk factors that may affect pedicle screw invasion into the proximal facet joint were selected and quantified: sex (male = 0, female = 1), age (< 50 years = 0, ≥ 50 years = 1), BMI (< 24 kg/m2 = 0, 24 ~ 28 kg/m2 = 1, > 28 kg/m2 = 2), bone mineral density (> 2.5 = 0, ≤ − 2.5 = 1), proximal facet joint angle (< 35° = 0, 35 ~ 45° = 1, > 45° = 2), degenerative lumbar spondylolisthesis (yes = 0, no = 1), isthmic lumbar spondylolisthesis (yes = 0, no = 1), and fixed segment (upper lumbar (L1-2) = 0, lower lumbar (L3-5) = 1).

### Statistical methods

SPSS 22.0 software (IBM, Armonk, NY, USA) was used for statistical analysis. Measurement data are expressed as the mean ± standard deviation. Count data were compared by the chi square test, *P* < 0.1. Logistic regression analysis of the factors that had statistical significance and were related to injury of the proximal facet joint caused by pedicle screw implantation was performed. *P* < 0.05 was considered statistically significant.

## Results

**Fig. 1:**
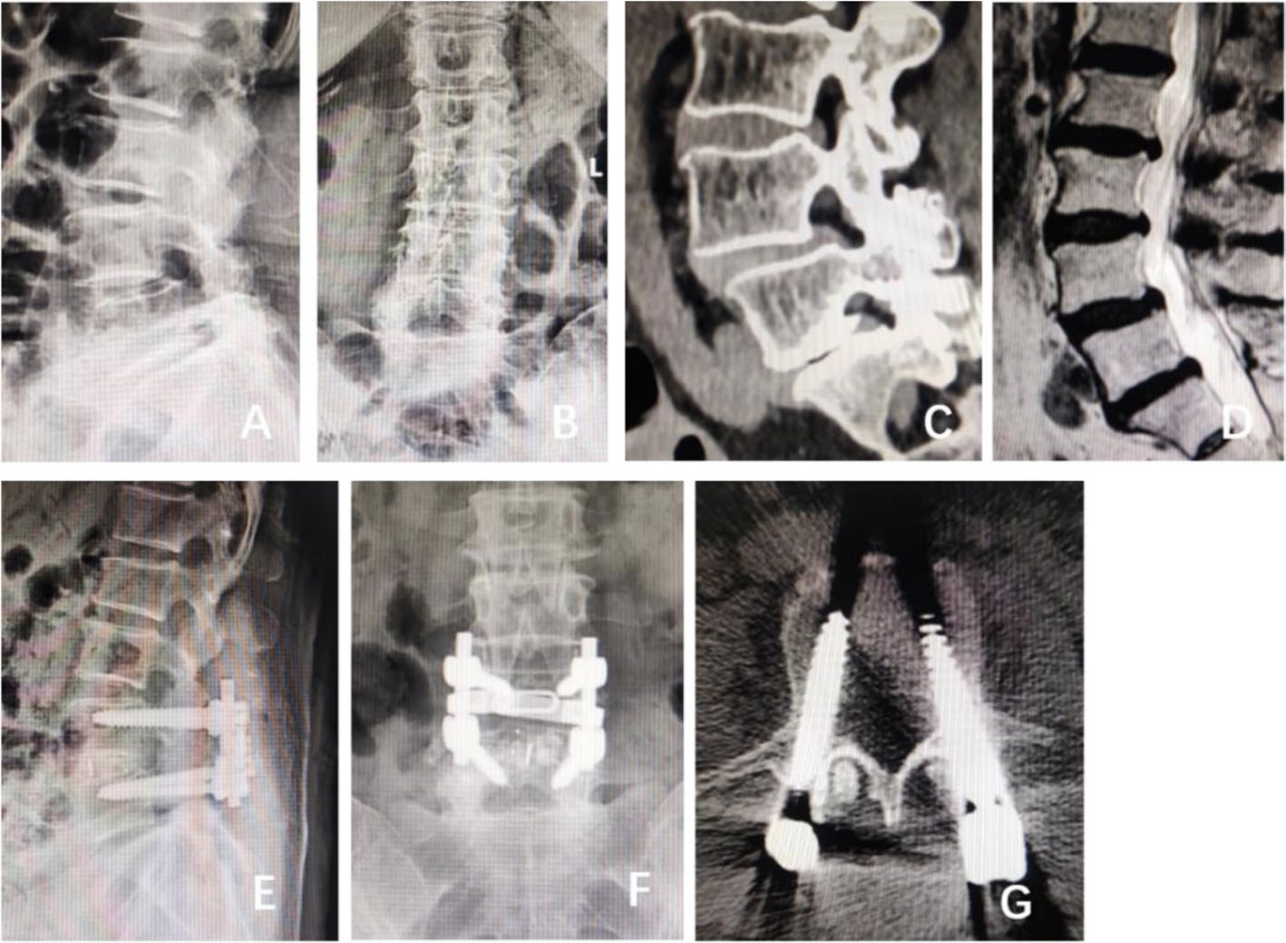
Li Moumou, a 63-year-old female, was diagnosed with degenerative lumbar spondylolisthesis and underwent posterior decompression, bone grafting, fusion and internal fixation at our hospital. **A**, **B**: L4 vertebral body spondylolisthesis (I°) before the operation. **C**: Preoperative CT showing lumbar degenerative changes and L4 spondylolisthesis (I°). **D**: Preoperative MRI showing L4 spondylolisthesis (I°), degeneration and bulging of L2-3, L3-4 and L4-5 intervertebral discs, and compression of the dural sac at the corresponding levels. **E**, **F**: X-ray showing the position of internal fixation of the lumbar spine 2 months after the operation. **G**: According to Herren’s grading method, the left screw showed no invasion of the proximal facet joint (grade 0); the right screw showed obvious entry of the articular cavity of the proximal facet joint (grade 2)

As shown in Table 1, there was a significant difference in age (*P* < 0.001), BMI (*P* < 0.001), proximal facet joint angle (*P* < 0.001), degenerative lumbar spondylolisthesis (*P* < 0.001), isthmic lumbar spondylolisthesis (*P* < 0.001) and fixed segment (*P* < 0.001) between the two groups (*P* < 0.05). There was no significant difference in sex or bone mineral density between the two groups (*P* > 0.05) Fig. 1.

**Table 1:** Comparison of each index between the two groups

Index	Number of cases	Invasion group (724 cases)	Noninvasion group (497 cases)	χ^2^	P
Sex				0.113	0.390
Male	612	360	252		
Female	609	364	245		
Age				60.441	0.000
< 50	481	230	251		
≥ 50	740	504	236		
BMI				71.745	0.000
< 24 kg/m^2^	393	211	182		
24 ~ 28 kg/m^2^	330	149	181		
> 28 kg/m^2^	498	364	134		
Bone mineral density				1.020	0.322
> −2.5	615	356	259		
≤ −2.5	606	368	238		
Proximal facet joint angle				117.078	0.000
< 35°	460	183	277		
35 ~ 45°	501	351	150		
> 45°	260	190	70		
Degenerative lumbar spondylolisthesis				47.921	0.000
No	550	268	282		
Yes	671	456	215		
Isthmic lumbar spondylolisthesis				114.587	0.000
No	370	133	237		
Yes	851	591	260		
Fixed segment				33.220	0.000
Upper lumbar	163	63	100		
Lower lumbar	1058	661	397

The indexes with a statistically significant difference on univariate analysis were included in logistic regression for multivariate analysis. As shown in Table 2, these indexes were age ≥ 50 years (*P* < 0.001, OR = 2.291), BMI > 28 kg/m2 (*P* < 0.001, OR = 2.548), degenerative lumbar spondylolisthesis (*P* < 0.001, OR = 2.187), isthmic lumbar spondylolisthesis (*P* < 0.001, OR = 2.410), proximal facet joint angle (35 ~ 45°: *P* < 0.001, OR = 3.151; 45°: *P* < 0.001, OR = 3.578), and fixed segment (lower lumbar spine: *P* < 0.001, OR = 2.912). This study considered that age (≥ 50 years old), BMI (> 28 kg/m2), proximal facet joint angle (35 ~ 45°, > 45°), degenerative lumbar spondylolisthesis, isthmic lumbar spondylolisthesis and fixed segment (lower lumbar spine) were risk factors affecting pedicle screw invasion of the proximal facet joint.

**Table 2:** Risk factor analysis

Index	B	SE	Wals	P	OR	95% CI	
						Lower limit	Upper limit
Age (≥50)	0.829	0.146	32.266	0.000	2.291	1.721	3.049
BMI			46.731	0.000			
BMI (24 ~ 28 kg/m^2^)	−0.169	0.179	0.884	0.347	0.845	0.595	1.201
BMI (> 28 kg/m^2^)	0.935	0.172	29.733	0.000	2.548	1.821	3.567
Proximal facet joint angle			66.542	0.000			
Proximal facet joint angle (35 ~ 45°)	1.148	0.160	51.757	0.000	3.151	2.305	4.308
Proximal facet joint angle (> 45°)	1.275	0.200	40.678	0.000	3.578	2.418	5.294
Degenerative lumbar spondylolisthesis (yes)	0.783	0.143	29.919	0.000	2.187	1.652	2.895
Isthmic lumbar spondylolisthesis (yes)	0.879	0.207	18.020	0.000	2.410	1.605	3.616
Fixed segment (lower lumbar)	1.069	0.143	56.212	0.000	2.912	2.202	3.851
Constant	−3.831	0.311	151.367	0.000	0.022		

## Discussion

### Screening of risk factors for pedicle screw invasion of the proximal facet joint

At present, the pedicle screw system is widely used in the treatment of spinal degenerative diseases; however, it is believed that the implantation of pedicle screws is an important cause of proximal facet joint injury [9]. In a previous report, Matsuzaki et al. [10] proposed for the first time that pedicle screws can invade the proximal facet joint and cause damage to the facet joint after lumbar surgery. Injury of the proximal facet joint will accelerate the degeneration of the facet joint, thus affecting the degeneration of segments adjacent to the fused vertebral body and the occurrence of degenerative diseases affecting the segments adjacent to the vertebral body and reducing the stability of the spine [6]. At the same time, damage to the facet joint tends to cause separation, placing greater pressure on the intervertebral disc of the adjacent segments [5]. This accelerates the degeneration of the segments adjacent to the intervertebral disc and thus accelerates postoperative adjacent segment degeneration (ASD). In the relevant literature, there have been few studies on the potential risk factors for proximal facet joint invasion in segments after unguided fixation. This study aimed to reduce the incidence of proximal facet joint invasion in unguided screw placement.

### Effect of lumbar spondylolisthesis on pedicle screw invasion of the proximal facet joint

The results showed that degenerative lumbar spondylolisthesis (*P* < 0.001, OR = 2.187) and isthmic lumbar spondylolisthesis (*P* < 0.001, OR = 2.410) increased the risk of postoperative pedicle screw invasion of the proximal facet joint. Relevant reports in the literature [11] have shown that a degree of lumbar spondylolisthesis > 10% is an independent risk factor for proximal facet joint injury. There is also a high probability of adjacent facet joint invasion after pedicle screw implantation during surgery. Therefore, spondylolisthesis of the responsible vertebral segment before surgery can easily lead to pedicle screw invasion of the proximal facet joint. Furthermore, isthmic spondylolisthesis is more likely to occur than degenerative lumbar spondylolisthesis. The reason may be that anterior lumbar flexion increases after degenerative vertebral spondylolisthesis, resulting in a deep surgical field, local structural disorder and difficulties during pedicle screw implantation, thereby increasing the likelihood of pedicle screw invasion of and damage to the proximal facet joint. Lumbar spondylolysis and spondylolisthesis are caused by fracture of the pedicle and hypertrophy and hyperplasia of the local vertebral facet, resulting in the formation of a large number of osteophytes and scar tissue; additionally, degeneration of the facet joint itself causes the facet joint to harden and deform, which can potentially result in the formation of a pseudojoint, changing the local normal anatomical structures and signs. To protect the joint capsule of the adjacent articular process during the operation, the position of the opposite screw point needs to be clear; otherwise, the pedicle screw can easily invade the proximal articular process after the operation. Therefore, while both degenerative lumbar spondylolisthesis and isthmic lumbar spondylolisthesis are independent risk factors affecting pedicle screw invasion of the proximal facet joint, pedicle screw invasion of the segmental facet joint is more likely in isthmic lumbar spondylolisthesis than degenerative lumbar spondylolisthesis.

### Effects of age, BMI, proximal facet joint angle and fixed segment on pedicle screw invasion of the proximal facet joint

The results of this study show that patient age ≥ 50 years (*P* < 0.001, OR = 2.262) and BMI > 28 kg/m2 (*P* < 0.001, OR = 2.516) increased the risk of proximal facet joint invasion. Matsukawa et al. [11] reported, greater osteophyte formation on local facet joints and local facet joint proliferation with increasing patient age; furthermore, because degeneration of the facet joint itself leads to hardening and deformation of the facet joint, resulting in changes in the local normal structures and landmarks, invasion of the proximal facet joint is more likely to occur during pedicle screw implantation. In obese patients, the elasticity of the paravertebral soft tissue may be very high, resulting in high pressure on local paravertebral soft tissue; this pressure could result in the compression and involvement of local soft tissue in the process of screw path preparation and screw placement, resulting in inward displacement and insufficient internal inclination [12, 13]. At the same time, local tissue hypertrophy increases the difficulty of exposing anatomical markers and increases the incidence of pedicle screw invasion of the proximal facet joint. However, some scholars believe that [14] young patients are more prone to facet joint injury caused by pedicle screws because the muscle fibers in the low back are developed and strong, with the potential to affect the accuracy of screw placement. Other scholars have proposed that [15] age has no effect on pedicle screw invasion of the proximal facet joint. In this study, age ≥ 50 years and BMI > 28 kg/m2 were independent risk factors for pedicle screw invasion of the proximal facet joint. However, the effect of age on pedicle screw invasion of the proximal facet joint is still controversial, and more studies with larger samples are needed for analysis and comparison.

The results of this study show that when the facet joint angle was 35 ~ 45° (*P* < 0.001, OR = 3.154) or > 45° (*P* < 0.001, OR = 3.585), the incidence of pedicle screw invasion of the facet joint increased significantly. The structure of the lumbar facet joint is complex. The direction of the joint surface gradually shifts to the coronal position from L1 to L5. The angle of the facet joint can be used as a quantitative parameter to reflect the structure of the lumbar facet joint. The facet joint angle was measured on the middle plane of the intervertebral disc and parallel to the superior endplate of the lower vertebral body. The angle between the line connecting the anteromedial point and the posterolateral point of the facet joint and the central line between the sagittal intervertebral disc and the bottom of the spinous process base is the lumbar facet joint angle [16]. In this study, the greater the angle of the facet joint, the more likely pedicle screw intrusion was after the operation. According to relevant reports in the literature [17], facet joint angles > 35° and > 45° are independent risk factors for facet joint injury. Referring to the relevant literature, we found the following reasons for these findings: ① during intraoperative positioning, the pedicle and facet joint may overlap under C-arm X-ray fluoroscopy, resulting in increases in the area of local screw placement and the incidence of facet joint injury; and ② because the articular surface of each facet joint of the lumbar spine gradually approaches the coronal position from top to bottom, screw placement can be affected and hindered by the articular surface and can even cause direct trauma to the articular surface and facet joint. Therefore, by measuring the facet joint angle of each patient and formulating a more optimized screw placement angle and direction before the operation, damage to the facet joint and the rate of pedicle screw invasion of the facet joint can be reduced. The results show that a facet joint angle of 35 ~ 45° or > 45° is an independent risk factor for pedicle screw invasion of the proximal facet joint. At the same time, when the fixed segment was in the lower lumbar spine (lower lumbar spine: *P* < 0.001, OR = 2.912), pedicle screw invasion of the proximal facet joint easily occurred. In a study by Tian et al. [18], from L1 to L5, the rate of proximal facet joint injury increased gradually. Moshirfar et al. [19] reported that the injury rate was highest after the placement of pedicle screws in the proximal facet joint of the L5 vertebral body compared with that of other lumbar bodies. We speculate that the reason for this finding is that with the decrease in the vertebral body level, the angle of the proximal facet joint will gradually increase, and the facet joint surface will gradually approach the coronal surface, which will lead to deviation of the screw entry point and screw entry channel. Therefore, pedicle screws easily invade the proximal facet joint in the lower lumbar spine. Other scholars have proposed that with greater lordosis of the lower lumbar spine, the placement of screws becomes increasingly blocked by the iliac crest, which may also increase the rate of damage to the proximal facet joint by pedicle screws [20]. Therefore, the segment of screw placement is an independent risk factor for pedicle screw invasion of the proximal facet joint. Manual screw placement was performed in all selected cases in this study, and although manual screw placement may lead to insufficient accuracy of the screw placement direction and angle, it is also easy for pedicle screw invasion of the proximal facet joint to occur with the application of corresponding equipment. However, Sakaura et al. [21] and Yson et al. [20] reported that invasion of the proximal facet joint can also occur during pedicle screw implantation using new technology and new auxiliary methods. Therefore, there is no clear superiority of pedicle screw placement by a manual or assisted method. However, the author believes that preoperative CT examination, pedicle morphology evaluation and facet joint angle measurement should be routine, and safe nail placement methods should be formulated for different patients. According to relevant literature reports, computer navigation and robot-assisted nail placement can reduce the risk of facet joint invasion. At present, there have been few relevant studies, and further clarification by large-sample and prospective research is needed.

### Limitations of this study

In this study, we did not analyze the risk factors for articular process injury at different levels or identify which risk factors caused the greatest or least damage to the proximal articular process joint. Additionally, the findings of this study need to be further confirmed by a prospective study with a large sample size.

## Conclusions

In conclusion, the results of this study show that age ≥ 50 years, BMI > 28 kg/m2, facet joint angle of 35 ~ 45° or > 45°, degenerative lumbar spondylolisthesis, isthmic lumbar spondylolisthesis and lower lumbar fixation are independent risk factors for pedicle screw invasion of the proximal facet joint after lumbar surgery. Furthermore, this study shows that while isthmic lumbar spondylolisthesis and degenerative lumbar spondylolisthesis are independent risk factors for pedicle screw invasion of the proximal facet joint after lumbar surgery, pedicle screw invasion of the segmental facet joint after lumbar surgery is more likely in isthmic lumbar spondylolisthesis.

## Supplementary Information

**Additional file 1.**

## Data Availability

All data and materials in this study are clinical data, which are available upon request. All data generated or analyzed during this study are included in this published article [and its supplementary information files].
